# First evidence for a possible invasional meltdown among invasive fish parasites

**DOI:** 10.1038/s41598-018-33445-4

**Published:** 2018-10-10

**Authors:** M. A. A. Hohenadler, K. I. Honka, S. Emde, S. Klimpel, B. Sures

**Affiliations:** 1 Aquatic Ecology and Centre for Water and Environmental Research, University of Duisburg-Essen, Universitätsstr. 5, 45141 Essen, Germany; 2 Present Address: Landesamt für Natur, Umwelt und Verbraucherschutz Nordrhein-Westfalen (LANUV), Fisheries Ecology, Heinsberger Str. 53, 57399 Kirchhundem-Albaum, Germany; 3 Goethe-University, Institute of Ecology, Evolution and Diversity; Senckenberg Gesellschaft für Naturforschung, Senckenberg Biodiversity and Climate Research Centre Frankfurt/Main, Max-von-Laue-Str. 13, 60438 Frankfurt, Germany; 4 Department of Zoology, University of Johannesburg, PO Box 524, Auckland Park, 2006 Johannesburg, South Africa

## Abstract

Biological invasions are frequently studied topics in ecological research. Unfortunately, within invasion ecology parasite-associated aspects such as parasite impacts on new environments and on local host populations are less well-studied. Round gobies migrating from the Ponto-Caspian region into the Rhine River system are heavily infested with the Ponto-Caspian acanthocephalan parasite *Pomphorhynchus laevis*. As shown by experimental infestations the acanthocephalans occur as pre-adults in host-encapsulated cysts within the internal organs of the migrating gobies, but remain infective for their definitive host chub. Recently, we described the occurrence of larvae of another parasite, the invasive eel swim bladder nematode *Anguillicola crassus*, in these *Pomphorhynchus* cysts. In the present study, we could prove the infectivity of the nematode larvae for European eels for the first time. After experimental inoculation of *Pomphorhynchus* cysts occasionally infested with *A*. *crassus* larvae, the nematodes grow to maturity and reproduce whereas all *P*. *laevis* were unviable. We therefore postulate that the nematode larvae behave like immunological hitchhikers that follow a “Trojan horse strategy” in order to avoid the paratenic host’s immune response. Accordingly, the interaction between both invasive parasites gives first evidence that the invasional meltdown hypothesis may also apply to parasites.

## Introduction

Invasion of free-living organisms and their effects on new habitats has emerged as a major threat for ecosystems around the globe, partly with irreversible consequences for the local biota. Invasive species might cause habitat modification, extinctions of endemic species, affect human health, and therefore engender enormous economic costs^1–3^. However, not every newly introduced species will be able to establish itself in a new habitat^4^. Success rates depend on different biotic and abiotic conditions such as absence/presence of enemies, competition with local species for resources, and climatic conditions^5–7^. Besides these aspects, the occurrence of other invasive species is one of the most substantial factors for invasion success. The so-called invasional meltdown hypothesis (IMH) states that if several new species invade the same habitat, they usually facilitate each other’s establishment since one species might serve e.g. as food or energy resource for another, which initiate its invasion process^8,9^. This might result in an increased rate of invasion, leading to crucial impacts within the new habitat^10^. In this context it seems surprising that alien parasites, although generally co-introduced to new environments with invasive host species^11–14^, are often not taken into account when evaluating the effects and mechanisms of invasion. This is even more surprising as parasites are considered an important response variable for ecosystem health^15–17^. Although the IMH did not show any significant differences among taxonomic groups that have been studied yet^18^ it remains unclear if it also applies to nonindigenous parasites.

In order to be able to invade a new habitat parasites usually depend on free-living alien hosts^19,20^. Therefore, the presence of a sufficient number of free-living invasive species is an obligate prerequisite for the establishment of non-indigenous parasites. Nevertheless, the question whether a certain parasite species also benefits from the occurrence of other invasive parasites remains to be unanswered. The Rhine River, a Western European river is considered a hot spot for biological invasion, and thus might be an ideal system to study the relevance of the IMH for invasive parasites^21,22^. Although many nonindigenous species were able to establish in the Rhine River over the past decades, invaders from water bodies of the Ponto-Caspian region were among the most successful^22^. Species such as the amphipod *Dikerogammarus villosus* or the fish species *Neogobius melanostomus* or *Ponticola kessleri* usually become dominant species in newly invaded areas due to their invasion strategy that provide them with competition advantages against local species^23,24^. Recent research has shown that both the amphipods as well as the fish species introduce the acanthocephalan *Pomphorhynchus laevis* to the river Rhine since the mid 1990’s after the inauguration of the Main-Danube-Canal^25^. Subsequently, the parasite spread rapidly and successfully established itself along the river Rhine, showing a high prevalence in cyprinid fishes as well as in predators that feed on infected intermediate host species^26,27^. After a potential paratenic host ingests pre-adult individuals of *P*. *laevis*, a cyst will be formed by both the hosts’ immune response and the parasite itself. Such parasite stages thus occur encapsulated in the hosts’ internal organs as well as in its body cavity^28–30^. Infection experiments with chub, *Squalius cephalus*, have demonstrated that encapsulation does not have any apparent effect on the parasite since it remains infective for its definitive host (unpublished data). Recent research has also shown that cysts of *P*. *laevis* in *N*. *melanostomus* may contain larvae of another invasive parasite species, *Anguillicola crassus*^31^. This nematode causes severe health impacts for the native eel species in Europe^32,33^. Initially it was co-introduced with Japanese eels (*Anguilla japonica)* to European waterbodies in the early 1980’s^34^. Shortly after its arrival, *A*. *crassus* adapted to local environmental conditions and accepted the European eel (*Anguilla anguilla*) as its suitable final host. Within a short period, the infestation rates of *A*. *crassus* in *A*. *anguilla* increased to more than 90% in large parts of Western and Central Europe (e.g.^33,35–37^). The nematode parasitizes the swim bladder of its final host after undergoing different development stages by using a wide variety of species as intermediate and paratenic hosts^38^. Accordingly, the eel’s swim bladder is frequently affected to a significant extent, leading to a reduced functionality, which might result in the host’s death during its spawning migration from the European coast to the Sargasso Sea^39^. In fact, *A*. *crassus* is also held partly responsible for the massive decline of the overall stock of European eel that resulted in its occurrence on the list of critically endangered species by the International Union for Conservation of Nature^40,41^.

The fact that individuals of *A*. *crassus* utilize cysts of encapsulated *P*. *laevis* individuals provides evidence that establishment of a parasite species might have been facilitated by the arrival of another invasive parasite within the Rhine River. Hyperparasitized cysts – what in detail describes acanthocephalan cysts that were simultaneously infested by *P*. *laevis* and *A*. *crassus -* which were gathered from *N*. *melanostomus* individuals from the Rhine River demonstrated that *A*. *crassus* frequently enters the cyst most likely to avoid immune responses of the paratenic host. Generally, third-stage larvae (L3) of *A*. *crassus* evoke an immune response of their paratenic hosts, with diversified intensities among the various host species, which might cause the parasites’ death^31^. Recently, it was suggested that *A*. *crassus* might use the cyst as a “hideout” to evade the immune response of the round goby, which might serve as prey for *A*. *anguilla*, the parasites’ main definitive host. Therefore, the nematode larvae are protected from host defenses while being in the goby. Theoretically, with such a “Trojan horse” strategy the parasite could be able to infest the hosts’ swim bladder more readily. However, it is still unknown whether *A*. *crassus* is still infectious for the definitive host after entering the acanthocephalan cyst. If yes, this could be seen as support that the IMH also applies to nonindigenous parasites. In order to test the viability and infectivity of encapsulated *A*. *crassus* larvae, we therefore conducted an infection experiment where European eels were inoculated with cysts collected from Ponto-Caspian gobies.

## Results

**Figure 1:**
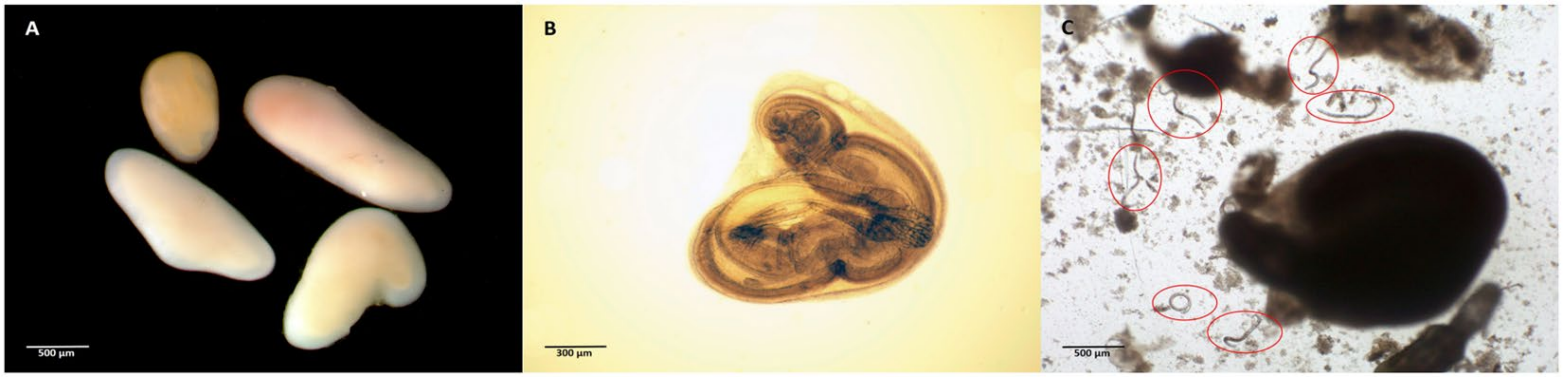
(**A**) Cysts of encapsulated *P*. *laevis* individuals as detected and removed from the digestive tracts of *N*. *melanostomus* (**B**) Encapsulated *P*. *laevis* irradiated with high light intensity (**C**) Digested cyst with released *A*. *crassus* individuals.

The initial screening of cysts removed from *N*. *melanostomus* (*cf*. Fig. 1) showed a prevalence of 12% of *A*. *crassus* larvae within the cysts. In all 200 cysts 96 larvae of *A*. *crassus* were detected, with a mean intensity of four nematodes per cyst (ranging between one to twelve larvae per cyst). Individuals of *P*. *laevis* found in the cysts were alive and showed a normal activity level.

Eels administered with intact cysts showed a prevalence with *A*. *crassus* of 40% 154 days post infection (dpi). While two eels were found to be infested by an individual *A*. *crassus* (either male or female) each, two eels showed a double infestation. In one eel two females occurred, whereas a pair of both sexes containing eggs with L2 larvae was detected in the second eel. In sum, 164 cysts were administered to the eels, which corresponds to a total of 79 *A*. *crassus* when considering the results of the initial cyst screening. Based on these results, the recovery rate can be determined as 7.6%. The size of the *A*. *crassus* individuals found in the eels corresponds with the developmental period of 154 days when compared with previous infection experiments^42,43^. Further parasitological examination of the eels did not show any infection with *P*. *laevis* in the experimental group. Eels of the uninfected control did not contain any individual of either parasite species.

## Discussion

The present study demonstrates for the first time that larvae of *A*. *crassus*, enclosed in the cysts of encapsulated *P*. *laevis*, remain able to infest their definitive host, the European eel. The experiment showed that *A*. *crassus* is still able to complete its life-cycle and produce offspring after entering the cysts in a potential paratenic host. Moreover, as the invasive nematode larvae use the cyst of an invasive acanthocephalan parasite species, the invasional meltdown hypothesis is supported.

Parasitological examination of the eels revealed a prevalence of 40% of *A*. *crassus* with a recovery rate of 7.6%. Previous experiments with eels using isolated L3 of *A*. *crassus* under similar conditions showed generally higher recovery rates of up to 40%^42,43^. Apart from the fact that the number of introduced *A*. *crassus* larvae in the present study can only be estimated as an average value and not using exact data, the relatively low infestation rate might also be related to this, so far unknown, way of transmission of *A*. *crassus*. It was demonstarted that encapsulation might be a barrier for some parasites in order to establish themselves after beeing transmitted to a new host^44^. As implied by the relatively low prevalence and recovery such a barrier effect might also apply for *A*. *crassus*. Nonetheless, the use of cysts containing encapsulated *P*. *laevis* in fish lacking a swim bladder represents an additional way of transmission to the preferred final host for *A*. *crassus*.

The results demonstrate the infectivity of *A*. *crassus* individuals from cysts co-infected with *P*. *laevis*. Thus, *A*. *crassus* was able to develop to mature adults whereas no individual of *P*. *laevis* was detected inside the eels at the end of the experiment although *P*. *laevis* is regularly found in eels from the Rhine River^25^. This is a striking result since encapsulated *P*. *laevis* that were ingested by their preferred definitive hosts such as *S*. *cephalus* and *Barbus barbus* are able to mature^28,44,45^, which was also confirmed by additional infection experiments in which encapsulated *Pomphorhynchus* individuals developed to full maturity after beeing infested to individuals of *S*. *cephalus* (unpublished data). The lack of any *P*. *laevis* in the examined eels after 154 dpi might therefore be related to the following reasons. On the one hand, the European eel as a non preferred host was used for laboratory infestation experiments. Even if *P*. *laevis* can regularly be found in eels in the field^21^ this might be a result of eels ingesting cystacanths from the first intermediate host, i.e. different species of amphipods and not by feeding on paratenic hosts. On the other hand, it is also conceivable that the lifetime of *P*. *laevis* in its non-preferred hosts is shorter than the time of seven to eight months estimated for this species in their preferred definitve hosts^46^. In the latter case, the acanthocephalans might have already been shed from the eels after 154 dpi. However, during daily inspections, no acanthocephalans were recovered in the tanks.

Both parasites have been described as succsessful invadors in European waterbodies and have been intensively studied during the past decades^47–49^. Nonetheless, a relation or possible interaction between the two invasive parasites was only discovered recently^31^. The reason might be that usually *P*. *laevis* is carefully removed from the cysts and then further examined while the tissue of the cyst is treated as waste material. Simultaneously, the larvae of *A*. *crassus* are not recognized since they are hardly seen by bare eye. Accordingly, the parasite has always been overlooked prior to the preliminary field study by Emde *et al*.^31^. Furthermore, we assume that if individuals of *A*. *crassus* have already been detected in gobies before, their exact localization (in the cysts) was not recognized. However, in the context of these findings and the results of the present study we assume that *P*. *laevis* might facilitate *A*. *crassus’* establishment and distribution in a new environment. This corresponds to the invasional meltdown hypothesis (IMH), which has never been described for invasive parasites before, although interactions of free-living invasive species are already referred to as a major aspect of biological invasion^9,18,50^. The IMH states that the arrival of nonindigenous species in an environment facilitates the establishment of other invasive species^8^. The fact that both parasites were able to establish themselves successfully in environments that are recognized as hotspots for invasion, such as the river Rhine, and the fact that *A*. *crassus* seems to benefit from the presence of encapsulated invasive parasites supports the assumption that the IMH also applies to invasive parasites.

Although *A*. *crassus* larvae utilize cysts and thereby eventually avoid the paratenic host’s immune response (of e.g. *N*. *melanostomus*) this could also be a side effect associated to the fact that gobies lack a swim bladder. It is already known that *A*. *crassus* larvae can be found in many different tissues of paratenic hosts^51–54^. The idea that the parasite uses a “Trojan horse strategy” was firstly mentioned in 2014^31^. Although the present results do not directly support a trojan horse strategy as no immunological responses were analysed, they show that *A*. *crassus* benefits from the presence of the cysts of encapsulated *P*. *laevis* individuals as it represents an additional way of infecting the definitive host. Obviously, the distribution and establishment of *A*. *crassus* is (at least partly) facilitated by another invasive parasite that consequently turned a possible dead-end host into a paratenic host in order to increase the nematodes’ infestation success. As there are not many other fish species described in which *P*. *laevis* occurs in cysts^29,55^, the particular type of co-occurrence of both parasites that is described here is only known for gobies.

The fact that both parasite species have been studied intensively over the past decades but their interaction was only discovered recently demonstrates the necessity of future research on possible interactions between (invasive) parasites in order to evaluate the effects of parasites invasion on local biota.

## Methods

A total of 22 individuals of the invasive goby *Neogobius melanostomus* were collected by professional fishermen with bow nets in the River Rhine close to the city of Grieth at Rhine km 844 (North Rhine Westfalia, Germany). Within two days after sampling, all fish were sacrificed and examined for the presence of acanthocephalans of the genus *Pomphorhynchus*, which were discovered encapsulated in the abdominal cavities of the fishes. All encapsulated *P*. *laevis* individuals (n = 364) were stored in a 0.9% sodium-chloride (saline) solution at 5 °C.

200 isolated cysts were transferred one by one into a well-plate chamber to check whether cysts were infested by *Anguillicola crassus*. Wells were filled up with artificial stomach acid-solution, composed of 1% hydrochloric acid and Pepsin (0.5 g per 100 ml)^56^. Filled-up well plates were incubated for 40 minutes at 37 °C to induce cysts to break open and allow parasites to be released and eventually found free in the solution (*cf*. Fig. 1C). After the incubation time, the content of each chamber was carefully examined in order to determine whether cysts were infested by *A*. *crassus* and if so, to what extent. The mean infestation rate (number of *A*. *crassus* per cyst of *P*. *laevis*) was calculated as 0.48, which was then used as a basis for subsequent infection experiments with European eels. We infested ten European eels (mean size of 426 mm) that were provided by a commercial eel farm known to be free of any infestation with *A*. *crassus* and/or *Pomphorhynchus* sp. with the remaining cysts (n = 164). Apart from a longstanding cooperation with the eel farm, eels are regularly checked by parasitological examinations to verify absence of *A*. *crassus* as well as of any other endoparasites. A total of 16 to 18 cysts (resembling approximately 7.7 to 8.6 *A*. *crassus*) were manually administered to each eel by a stomach tube (diameter of 0.5 mm). Following infection, the eels were kept individually in a single water tank (30 l) at a water temperature between 10 and 13 °C with permanent air supply. An uninfected control group of five eels (mean size of 464 mm) was kept under the same experimental conditions to verify that the eels were free of parasites. The eels were killed and examined for parasites 154 days post infection (dpi). Internal organs were removed and digestive tracts and swim bladders were carefully examined under a stereomicroscope for the presence of *A*. *crassus* and *P*. *laevis*. Individuals of *A*. *crassus* were subsequently categorized according to their developmental stage and sex.

All experimental protocols were approved by the Ethics Council (Landesamt für Natur, Umwelt und Verbraucherschutz, Nordrhein-Westfalen, permit number: 84-02.04.2017.A245) and were carried out in accordance with the relevant guidelines and regulations.

## Data Availability

The datasets generated during and/or analysed during the current study are available from the corresponding author on reasonable request.
